# Topological polymorphism of nucleosome fibers and folding of chromatin

**DOI:** 10.1016/j.bpj.2021.01.008

**Published:** 2021-01-16

**Authors:** Victor B. Zhurkin, Davood Norouzi

**Affiliations:** 1 Laboratory of Cell Biology, Center for Cancer Research, National Cancer Institute, National Institutes of Health, Bethesda, Maryland

## Abstract

We discuss recent observations of polymorphic chromatin packaging at the oligonucleosomal level and compare them with computer simulations. Our computations reveal two topologically different families of two-start 30-nm fiber conformations distinguished by the linker length L; fibers with L ≈ 10n and L ≈ 10n+5 basepairs have DNA linking numbers per nucleosome of *Δ*Lk ≈ −1.5 and −1.0, respectively (where n is a natural number). Although fibers with *Δ*Lk ≈ −1.5 were observed earlier, the topoisomer with *Δ*Lk ≈ −1.0 is novel. These predictions were confirmed experimentally for circular nucleosome arrays with precisely positioned nucleosomes. We suggest that topological polymorphism of chromatin may play a role in transcription, with the {10n+5} fibers producing transcriptionally competent chromatin structures. This hypothesis is consistent with available data for yeast and, partially, for fly. We show that both fiber topoisomers (with *Δ*Lk ≈ −1.5 and −1.0) have to be taken into account to interpret experimental data obtained using new techniques: genome-wide Micro-C, Hi-CO, and RICC-seq, as well as self-association of nucleosome arrays in vitro. The relative stability of these topoisomers is likely to depend on epigenetic histone modifications modulating the strength of internucleosome interactions. Potentially, our findings may reflect a general tendency of functionally distinct parts of the genome to retain topologically different higher-order structures.

## Significance

Recent advances in superresolution cell imaging techniques and genome-wide analyses of the nucleosome interaction frequencies have revealed highly variable configurations of chromatin fibers. These observations are accompanied by multiscale computational modeling providing valuable structural information. Here, we demonstrate that these computations offer semiquantitative interpretations of recent experiments and bring new insights into the interplay between the local folding motifs, the global topological polymorphism of chromatin, and the level of transcription.

## Introduction

According to the canonical textbook model of eukaryotic chromosomes (1), DNA undergoes several rounds of hierarchical packaging, starting with linear chains of nucleosomes (“beads-on-a-string”) that are further folded in the solenoid-like 30-nm fibers (2), chromatin 300-nm loops, and so on, up to chromosome territories in the nucleus. The solenoid model was inconsistent with a significant body of data (3, 4, 5, 6), yet it was widely accepted in the field. Over the past few years, however, we have been witnessing a paradigm shift in the way chromatin folding is described (for reviews, see (7,8)).

Because of significant progress in experimental techniques, such as superresolution cell imaging (9, 10, 11, 12) and electron microscope tomography (13), it became clear that in the native state, nucleosome chains are assembled in a diverse and disordered manner. Recent analyses of the nucleosome interaction frequencies using genome-wide Micro-C (14, 15, 16), Hi-CO (17), and RICC-seq (18) revealed several distinct orientations of adjacent nucleosomes generally supporting the two-start (zigzag) organization of chromatin fibers. Thus, instead of a regular 30-nm solenoid (2) hierarchically coiled in the higher-order structures, now we envision irregular folding of nucleosomes in variable two-start fiber configurations, which, in turn, form liquid-like condensates inducing phase separation between the hetero- and euchromatin (19,20). At the megascale end of DNA packaging in vivo, various chromosome conformation capturing techniques (e.g., Hi-C (21,22)) have detected formation of large DNA loops and topological domains stabilized by nucleoprotein complexes including CTCF and cohesin (in interphase chromosomes) or condensin (in metaphase). This multilayer organization of DNA is highly dynamic, which is essential for all DNA-related biological processes as it helps DNA overcome numerous conformational and topological constraints during the cell cycle.

Conformational dynamics of chromatin fibers has been extensively analyzed using multiscale computational modeling (reviewed in (23,24)). These studies revealed pronounced structural polymorphism of fibers triggered by variations in DNA linker lengths or other parameters, such as linker histone densities and distribution of acetylation marks (25, 26, 27, 28, 29). Application of electron microscopy-assisted nucleosome interaction capture (EMANIC) cross-linking experiments in combination with mesoscale modeling allowed us to observe hierarchical loops (or loops of loops) leading to enhanced long-range interactions between nucleosomes (30). Recent modeling suggested that cooperation between epigenetic factors can facilitate hierarchical looping in an ∼50-kb gene cluster (31). It would be interesting to compare these results with the superresolution imaging data (11,13).

Much less is known about the global topological polymorphism of DNA in eukaryotic chromosomes. Only a limited number of theoretical studies (32, 33, 34, 35, 36) was devoted in the past decade to topological diversity of chromatin fibers, in which the DNA linking number and writhing (37,38) were related to the epigenetic state of the nucleosome and/or the configurations of nucleosome arrays.

Below, we discuss recent findings related to the topological aspects of chromatin packaging and demonstrate that the observed polymorphism of DNA folding can be interpreted based on rigorous computations of nucleosome fibers. New evidence is presented for the existence of two distinct families of fiber topoisomers characterized by different nucleosome spacing.

### Topological polymorphism of chromatin fibers

Soon after the nucleosome was discovered (39, 40, 41), it became obvious that there is a discrepancy between nucleosome structure and DNA topology in the SV40 minichromosome (the so-called “DNA linking number paradox” first formulated by Crick (42) and Fuller (43)). This conundrum has been described many times during the past 40 years (44,45), so we can skip technical details. In short, measurements of DNA topology in circular minichromosomes showed generation of only one negative superhelical turn per nucleosome (46,47), instead of 1.6–1.7 negative turns expected from the left-handed DNA wrapping in the nucleosome.

The DNA linking number, Lk, defines the number of times each DNA strand winds around the other. The DNA writhing, Wr, characterizes trajectory of the DNA axis in space. For closed circular DNA, the change in the linking number, *Δ*Lk (compared with the relaxed state of DNA), the change in DNA twisting, *Δ*Tw, and DNA writhing, Wr, are related by the well-known equation *Δ*Lk = *Δ*Tw + Wr (37,38).

To explain the above paradox, several models of the 30-nm fiber, with *Δ*Lk = −1 and −2, were suggested in the 1980s (48, 49, 50). They remained untested for ∼20 years until the first crystal structure of the tetranucleosome was solved by Richmond and associates (51). This structure, as well as the cryo-electron microscopy (Cryo-EM) and x-ray fiber conformations published later (52), were obtained for tandem repeats of strongly positioned nucleosomes “601” (53). (Note that *Δ*Lk ≈ −1.5 for these fibers.)

The linking number paradox still remained unresolved, however, because in all mentioned fiber structures, the internucleosomal linkers contain the integral numbers of DNA turns (linker length L = 20, 30, and 40 basepairs (bp), denoted here as {10n}), whereas biochemical (54,55) and genomic studies (56, 57, 58) show the prevalence of linkers with half-integral numbers of DNA turns (denoted as {10n+5}). Therefore, it was critically important to clarify whether chromatin fibers with {10n} and {10n+5} linkers fold differently as this might be a key to resolve the *Δ*Lk paradox.

To address this issue, we have systematically analyzed stereochemically feasible two-start fibers, with the linkers varying in a wide interval from 10 to 70 bp (33,34). In addition to the DNA linker length L, the regular chromatin fibers are characterized by the nucleosome repeat length (NRL) and the DNA length in nucleosome core particle (L_NCP_), such that NRL = L_NCP_ + L. Usually, it is assumed that L_NCP_ = 147 bp, which is consistent with the highest-resolution x-ray structure of the nucleosome particle (59). However, in the case of strongly positioned nucleosome “601” selected by Lowary and Widom (53), we are using L_NCP_ = 146 bp, in accordance with the crystal structures of this nucleosome core particle (60). To keep DNA distortions within the limits observed in x-ray structures, we calculated the elastic energy of DNA deformations using knowledge-based potential functions (61).

**Figure 1:**
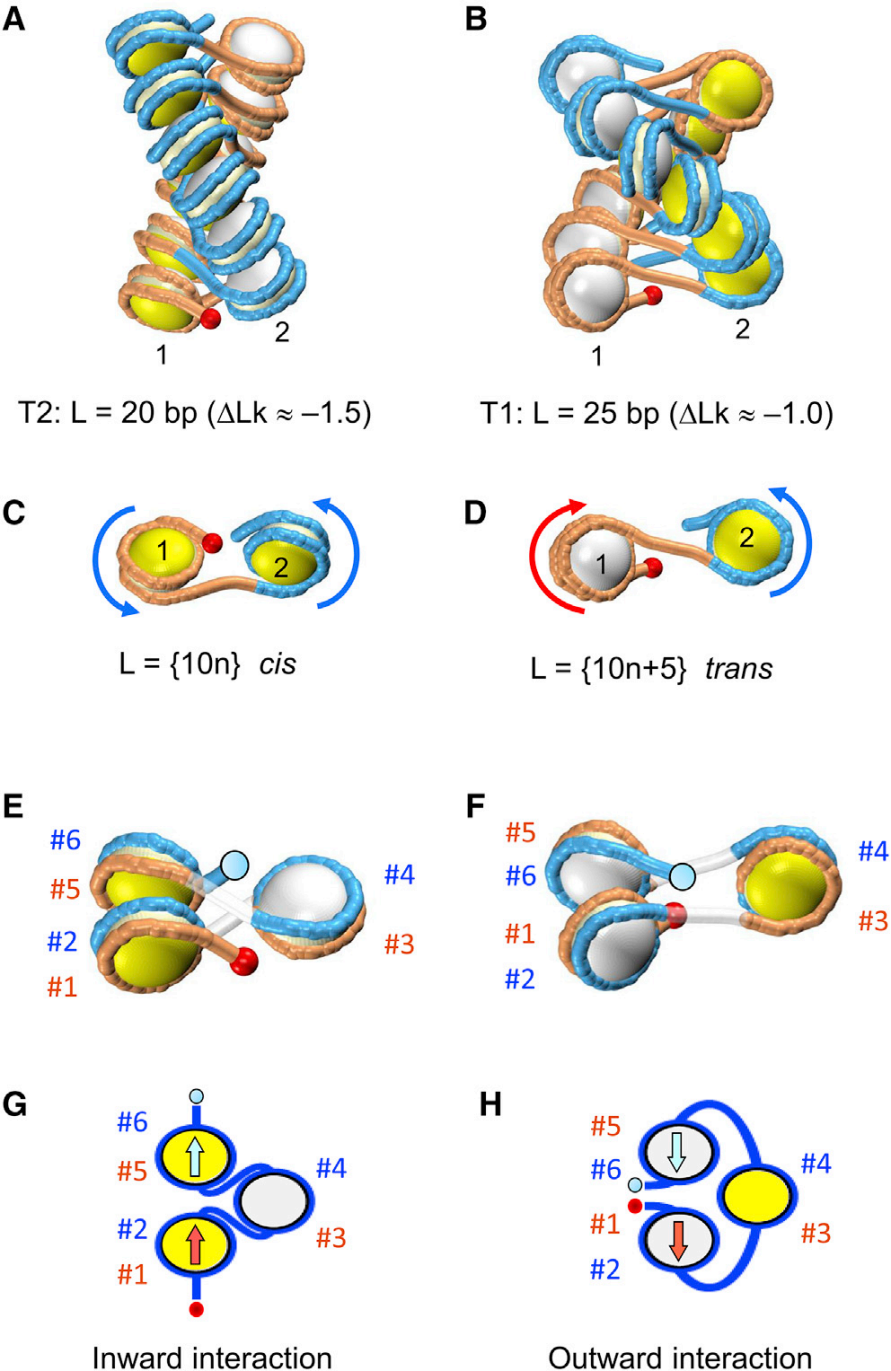
Distinctive “rotational setting” of adjacent nucleosomes in the T2 and T1 topoisomers produces different conformations of the nucleosome arrays. (*A* and *B*) Shown are energetically optimal two-start fibers with free DNA linker lengths L = 20 and L = 25 bp, respectively (33). Computations were made for L_NCP_ = 147 bp (59). Note that the red sphere (indicating the “entry” point) is positioned differently in the two fibers; in (*A*), it is facing the viewer, whereas in (*B*), it is pointed away from the viewer. In each nucleosome, the entry side is colored in yellow and the exit side in white. (*C* and *D*) Shown are the differences in the DNA pathway of successive nucleosomes (here 1 and 2) of the fibers in (*A* and *B*). (*C*) Bottom view, L = 10n is shown. Both nucleosomes face the viewer from the yellow side, and the arrows indicating the DNA trajectory are directed similarly, counterclockwise. The DNA linker contains (approximately) an integral number of helical turns of DNA; thus, the two adjacent nucleosomes are in a *cis*-like configuration. (*D*) Front view, L = 10n+5 is shown. Note the different orientations of nucleosomes 1 and 2 that face the viewer from the white and yellow sides, respectively. The arrows run clockwise (nucleosome 1) and counterclockwise (nucleosome 2). Changing the linker DNA length by 5 bp introduces an additional half-turn of the DNA duplex, resulting in a *trans*-like configuration of the two nucleosomes. (*E* and *F*) The entry and exit halves (gyres) of nucleosomes are colored differently to emphasize the distinctive spatial organization of DNA in the T2 and T1 topoisomers. As above, the red spheres indicate the “entry” points and the light blue circles the “exit” points of the trinucleosomes. In (*E*), gyres #2 and #5 are in contact; we call this the T2 fold. In (*F*), gyres #1 and #6 are in contact; this is the T1 fold. (*G* and *H*) The two types of internucleosome interaction in yeast chromatin observed by Ohno et al. (17) closely correspond to the folding motifs presented in (*E* and *F*). Compare the positioning of the “entry” and “exit” points in (*E* and *G*) on the one hand and in (*F* and *H*) on the other hand. Furthermore, the arrangement of gyres (#1, #2, #5, and #6) is also the same for (*E* and *G*) (in the *left column*) and for (*F* and *H*) (in the *right column*). According to notations used by Ohno et al. (17), the internucleosome contacts shown in (*G* and *H*) are denoted “inward” and “outward” interactions, respectively (see their Fig. S1, D and G (17)). Note that the “inward” and “outward” interactions (17) correspond to the IN-IN and OUT-OUT orientations of nucleosomes (according to the notations of Hsieh et al. (14)). To see this figure in color, go online.

As a result, we discovered, in silico, two distinct families of fiber conformations (T2 and T1) with different DNA topologies. One family, T2, is represented by topoisomers similar to the fibers observed in crystals (51,52), whereas the other family, T1, contains novel forms with a different DNA folding (Fig. 1, *A* and *B*). Importantly, the topology of a chromatin fiber strongly depends on nucleosome spacing; the energetically optimal fibers with the {10n} and {10n+5} linkers have DNA linking numbers per nucleosome *Δ*Lk ≈ −1.5 and −1.0, respectively. This topological polymorphism can be utilized in vivo for gene regulation.

We obtained this result (33,34) evaluating the Wr and *Δ*Lk values through numeric computations of the Gauss double integral (62,63). At the qualitative level, it is illustrated in Fig. 1, *C* and *D*, where the difference in *Δ*Lk between the T2 and T1 topoisomers is reduced to alteration of the rotational setting of adjacent nucleosomes by 180°, which is a consequence of changing the DNA linker length by 5 bp, from {10n} to {10n+5}. The change in the linker DNA twisting by 180° corresponds to the *ΔΔ*Lk ≈ 0.5 mentioned above; therefore, intuitively, we can link the topological difference between the T2 and T1 forms to the *cis*- to *trans*-like transition shown in Fig. 1, *C* and *D*. In addition, we emphasize distinct interactions between the entry and exit halves of the nucleosomes in the T1 and T2 topoisomers (Fig. 1, *E* and *F*). This difference is essential for interpretation of the results obtained in radioprobing studies of DNA folding in chromatin (18) and in high-resolution Hi-CO (17) and Micro-C (14, 15, 16) experiments performed recently (see below).

The predicted topological polymorphism of chromatin fibers (33,34) was confirmed experimentally (64) by employing topological gel assays and electron microscopy imaging. Using circular DNA constructs with regularly positioned nucleosomes “601,” we demonstrated that the nucleosome arrays with NRL = 167 and 172 bp are characterized by *Δ*Lk = −1.4 and −0.9, respectively (64). In other words, the DNA supercoiling changes by as much as 50%, depending on the length of the DNA linker between nucleosomes, in excellent agreement with theoretical results (33). This observation was made for relatively short 20- and 25-bp linkers observed in yeast (57,58). Recently, we corroborated this conclusion analyzing nucleosome arrays with 182- and 187-bp NRL (65), typical for higher eukaryotes (L = 36 and 41 bp).

Thus, we made an important step toward resolving the long-standing linking number paradox. We have proven that there is no single *Δ*Lk value characterizing ensembles of various chromatin fiber configurations in general. In fact, the average linking number is defined by nucleosome spacing (and therefore by NRL) and varies at least from −0.9 to −1.4. (According to our computations (33,34), *Δ*Lk varies from −0.8 to −1.7 in the energetically feasible regular conformations.) The value *Δ*Lk = −1.26 measured recently for the yeast minichromosomes by Segura et al. (45) fits in this interval and reflects the average *Δ*Lk for those configurations of the relatively short nucleosome chains that were stabilized under the experimental conditions.

### Nucleosome spacing and the level of transcription

The topological polymorphism of chromatin fibers described above may play a role in the regulation of transcription. According to the model of Liu and Wang (66), the level of negative supercoiling of DNA is decreased downstream and increased upstream of the transcription complex. Therefore, we hypothesized that the existence of the two types of fibers (T1 and T2) with different linking numbers may be related to the transient DNA topological changes occurring during transcription. We reckoned that the T1 topoisomer with *Δ*Lk ≈ −1 (and a weak supercoiling of DNA) would be formed predominantly downstream from RNA polymerase (in the highly transcribed genes), as opposed to the T2 topoisomer with *Δ*Lk ≈ −1.5, which is likely to be stabilized in the upstream regions (and more generally, in the regions with a low level of transcription).

Because the T1 and T2 topoisomers are characterized by distinct linker lengths, {10n+5} and {10n}, respectively, we expected to see a difference in the distribution of the sizes of internucleosome linkers in highly and lowly expressed genes. To verify this assumption, we compared the nucleosome positions in the yeast genes from the top and bottom 25% of the expression level scale (34). Indeed, the two sets of genes were found to have different distributions of nucleosome repeats: for the highly expressed genes, NRL ≈ 161 bp (the average linker length <L> = 14 bp), and for the lowly expressed genes, NRL ≈ 167 bp (<L> = 20 bp).

These results are consistent with the above hypothesis that nucleosome arrays with L ≈ 10n+5, which have a relatively low superhelical density, are transcriptionally more competent than the arrays with L ≈ 10n. In addition, the fibers with L ≈ 10n+5 reveal a greater plasticity (33), which may facilitate the formation of gene loops (67) and enhancer-promoter loops (68), thereby further inducing transcription of the corresponding genes. By contrast, in inactive genes, the prevalent linker length is L ≈ 10n, which corresponds to a higher superhelical density and a higher stability of the chromatin fiber.

In higher eukaryotes, the genomic organization is more complicated than in yeast, and simple classification of genomic regions in two categories, active and repressed, is not feasible. Instead, one has to consider at least half a dozen types of nuclear domains (compartments) characterized by distinct chromatin epigenetic states, two of which represent active chromatin and the other four represent repressed chromatin (69). Available data suggest that there are certain correlations between the DNA linker lengths and epigenetic modifications (70,71), which are essential for gene regulation. In particular, the H3K9-methylated constitutive heterochromatin regions in *Drosophila* have average linker length <L> = 30 bp, or {10n}, whereas the polycomb-repressed H3K27-methylated chromatin has <L> = 26 bp (71). Notably, the highly expressed genes display the shortest linkers, with average <L> = 17 bp—that is, close to {10n+5} values.

Overall, we see that the link between the level of transcription and the nucleosome spacing, described above for the yeast genes, is valid for the *Drosophila* genes as well, but the correlation is weaker in this case.

Very recently, the idea on the interplay between nucleosome spacing, gene expression, and chromatin epigenetic state gained strong (albeit indirect) support by the in vitro observation (19) that the chromatin fibers’ ability to form phase-separated liquid condensates drastically differs for arrays with DNA linker lengths belonging to the series {10n} and {10n+5}. Below, we show that this tendency can be explained by the distinct conformational variability of the two types of nucleosome arrays.

### Conformational dynamics of chromatin fibers and nucleosome spacing

**Figure 2:**
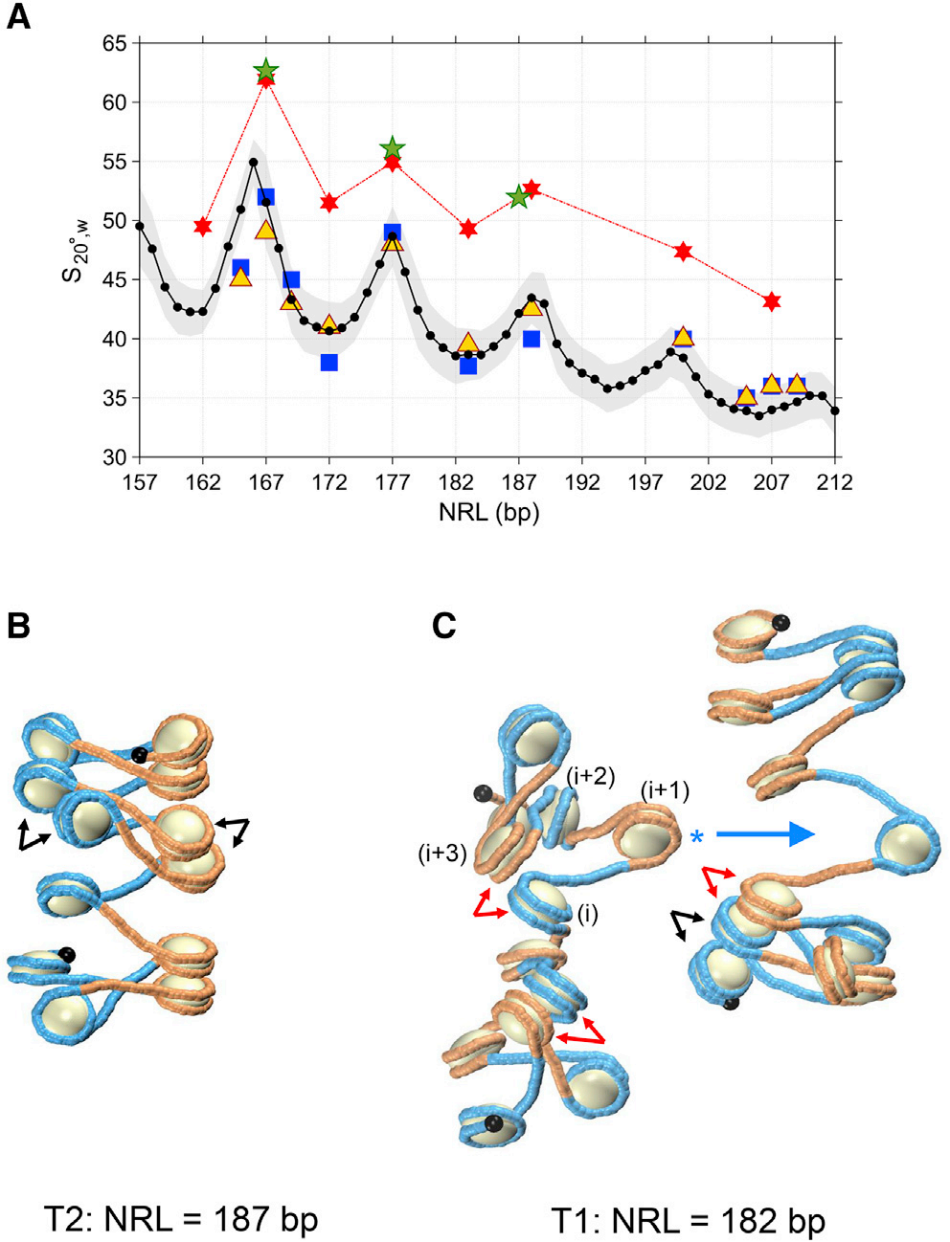
Compactness of nucleosome array folding depends on NRL. (*A*) Predicted sedimentation coefficient (s_20°,w_) was calculated for MC ensembles of 12-mer arrays of positioned nucleosomes “601” with L_NCP_ = 146 bp (60). Average MC values are shown by the black solid line, with standard deviations indicated by the gray area (65). The blue squares and yellow triangles represent the experimental values obtained at 1 mM MgCl_2_ and 150 mM NaCl, respectively (65,72). The red six stars are for the energetically optimal fiber conformations (33) and the green five stars are for the x-ray (51) and Cryo-EM (52) fiber structures, which belong to the T2 family, with L ≈ {10n}. (*B* and *C*) Shown are typical MC conformations (65,73) obtained for fibers with NRL = 187 bp (*B*) and 182 bp (*C*). Note that the 187 × 12 structure is more compact than the 182 × 12 structures, in agreement with sedimentation experiments (*A*). Close contacts between nucleosomes are indicated by black (i, i±2) and red arrows (i, i±3). The flipped-out nucleosome (i+1) is marked by an asterisk (*C*). This nucleosome can interdigitate in the neighboring nucleosome array (*large blue arrow*). In the fiber with NRL = 187 bp (*B*), all close contacts are of the (i, i±2) type (shown by *black arrows*), in agreement with EMANIC data; the nucleosome flipping out occurs much less frequently than for NRL = 182 bp (65). To see this figure in color, go online.

Sedimentation experiments performed in Grigoryev’s group (65,72) and our Monte Carlo (MC) simulations (73) reveal periodic modulation of nucleosome array folding upon an increase in linker DNA length (Fig. 2 *A*). Importantly, both measurements and calculations of the sedimentation velocity (74) were performed for 12-mer arrays of positioned nucleosomes “601” (53). The highest sedimentation coefficients (and the most compact array folding) are observed for L ≈ 10n. By contrast, the lowest sedimentation (and the “loosest” fiber folding) are observed for L ≈ 10n+5; see typical MC conformations in Fig. 2, *B* and *C*. Notably, these distorted fibers are significantly more extended than the regular structures presented in Fig. 1, *A* and *B*. This feature is critical for reproducing the experimentally observed hydrodynamic properties of the nucleosome arrays. Otherwise, consideration of compact regular configurations obtained either computationally (33) or experimentally under cryogenic conditions (51,52) leads to an overestimation of the nucleosome fiber stiffness in solution (72,73); see the *red* and *green stars* in Fig. 2 *A*.

Note that the MC-induced thermal fluctuations lead to a different loss of nucleosome stacking in fibers from the T1 and T2 families (Fig. 2 *C*). The fraction of stacked nucleosomes for the T1 fibers is ∼30% as compared with ∼60% for the T2 fibers (73). Therefore, we expect that the free energy balance between the T1 and T2 conformers depends on the factors modulating internucleosome interactions. In particular, the H4-K16 acetylation destabilizing the nucleosome stacking is likely to increase the probability of formation of the T1 conformer.

The more pronounced variability of the T1 conformers (Fig. 2 *C*) allows for interpreting the recent finding of an extensive self-association of nucleosome arrays in the case of {10n+5} but not {10n} linkers (19). The T1 forms are characterized by frequent contacts between nucleosomes (i) and (i±3), in addition to the (i, i±2) contacts typical of the two-start configurations (see *black* and *red arrows* in Fig. 2 *C*). This tendency is entirely consistent with the data obtained by EMANIC (30,65,75). Importantly, the (i, i+3) interaction is accompanied by the flipping of nucleosome (i+1) out of the fiber (see *asterisk* in Fig. 2 *C*). This configuration is perfectly suitable for interdigitating the flipped-out nucleosome (6,76) into the neighboring array (*large blue arrow* in Fig. 2 *C*) and thus stabilizing macroscopic aggregates observed by Gibson et al. (19).

By contrast, the (i, i±2) stacking interactions are predominant in the T2 fibers with L ≈ 10n (Fig. 2 *B*). Flipping out of nucleosomes is very rare in this case as well as formation of the (i, i±3) contacts (30,65,73). This explains the observation (19) that fibers with {10n} linkers are much less prone to self-association, at least in the absence of nonhistone proteins such as HP1 (20).

To summarize, our MC simulations of isolated oligonucleosomal arrays (65,73) account for the observed difference in the propensity of {10n+5} and {10n} fibers for macroscopic self-association (19). This difference is likely to be a key to understanding the basic molecular mechanisms of stabilization of nuclear compartments formed by active and repressed chromatin.

### Radioprobing DNA folding in situ and topological state of chromatin

**Figure 3:**
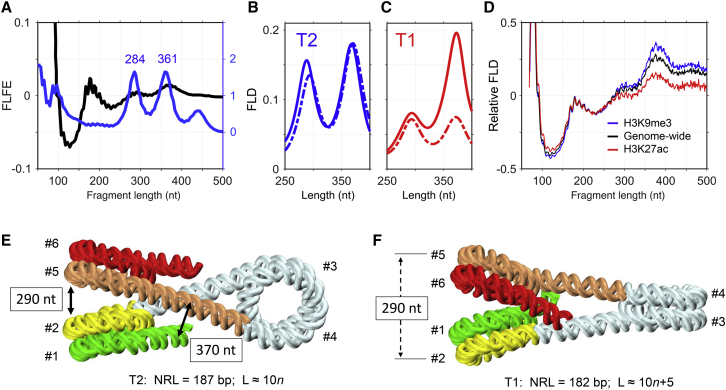
Comparison of the experimental RICC-seq data with theoretical predictions. (*A*) The experimental genome-wide FLFE profile (18) is shown by the black curve. The blue curve corresponds to the FLD calculated for the Cryo-EM tetranucleosome structure with NRL = 187 bp (47) (based on Fig. S10 g from Risca et al. (18)). (*B* and *C*) Shown are the calculated FLD profiles for the topoisomers T2 (NRL = 187 bp, in *blue*) and T1 (NRL = 182 bp, in *red*). Computations were made for L_NCP_ = 147 bp (59). The FLD profiles for energetically optimal structures are shown in solid lines and for the MC ensembles in dashed lines. The data are presented for the interval 250–400 nt; beyond these limits, the FLD values are close to those shown in (*A*) (*blue curve*). To calculate FLD (n) for a given fiber conformation, for each fragment of length “n” bp, frequency of correlated DNA breaks at the ends of the fragment was calculated, and then all such frequencies were averaged (for each “n” separately). The frequency of DNA breaks as a function of three-dimensional distance between the fragment ends decreases exponentially, with the exponential drop constant *λ* = 4.0 nm (18). (*D*) Shown are the FLD profiles calculated for the transcriptionally active (H3K27ac, *red curve*) and repressed (H3K9me3, *blue curve*) regions in the human genome, based on the RICC-seq data by Risca et al. (18). The genome-wide distribution is shown by the black curve. (*E* and *F*) Distinct DNA folding in the T2 and T1 topoisomers leads to different FLD profiles. The entry and the exit halves of nucleosomes (gyres) in the left stack are colored differently to emphasize the different spatial organization of DNA in the two cases. Whereas in the case of NRL = 187 bp (*E*), the yellow (#2) and light brown (#5) gyres separated by 290 nt are spatially close, the same gyres #2 and #5 are spatially distant in the case of NRL = 182 bp (*F*). This explains why the 290-nt peak is stronger for NRL = 187 bp (*B*). Note that in (*E*), the gyres in the left stack are positioned in the order #1, #2, #5, and #6, which is the same as in Fig. 1*E*. By contrast, the order is #2, #1, #6, and #5 in (*F*) and in Fig. 1*F*. To see this figure in color, go online.

Risca et al. (18) used ionizing RICC-seq to identify the DNA-DNA contacts that are spatially proximal in situ in human cells. The experimentally observed fragment length fold enrichment (FLFE) profile was compared to the fragment length distribution (FLD) computed for the DNA folding in the 30-nm fiber (52) (see Fig. 3 *A*). Positioning of the FLFE local maxima at 280–290 and 360–370 nucleotides (nt) are in line with the zigzag folding of DNA, as was shown earlier by Rydberg et al. (77). Note, however, that the equal heights of the 284-nt and the 361-nt peaks in FLD computed for the DNA folding in the 30-nm fiber (52) are inconsistent with the FLFE profile (compare *black* and *blue lines* in Fig. 3 *A*). It is therefore unlikely that RICC-seq data can be interpreted based solely on the crystal structures of chromatin fibers, and some alternative fiber conformations should be taken into account. We suggested that the novel T1 topoisomer described above could be such an alternative (78).

To test this hypothesis, we calculated FLDs for the T1 and T2 conformers using the same assumptions as used by Risca et al. (18)—namely, that frequency of correlated breaks in DNA exponentially depends on three-dimensional distance between the points of cleavage (Fig. 3, *B* and *C*). We found that FLD profiles for the energetically optimal and MC-simulated T2 forms with NRL = 187 bp are similar to that calculated by Risca et al. (18) for the Cryo-EM tetranucleosome structure (52), also with NRL = 187 bp (Fig. 3 *A*), which is not surprising because these conformations are topologically equivalent. On the other hand, for the energetically optimal T1 topoisomer, the 290-nt peak is significantly lower than the 370-nt peak (Fig. 3 *C*, NRL = 182 bp). This is explained by different folding of DNA in the T1 and T2 fibers (Fig. 3, *E* and *F*).

In the T2 topoisomer (Fig. 3 *E*), the exit of the first nucleosome (gyre #2) is spatially close to the entry of the adjacent nucleosome (gyre #5). By contrast, in the T1 topoisomer (Fig. 3 *F*), gyres #2 and #5 are spatially distant, although in both cases they are separated by ∼290 nt on average. Hence, the 290-nt peak is weaker for the T1 conformer, which is generally consistent with the asymmetric FLFE profile presented in Fig. 3 *A*.

The other strong peak in the FLD profiles, at 370 nt, reflects close proximity of the histone-free linkers (Fig. 3 *E*). This peak is significantly weaker for the MC-simulated T1 fibers (Fig. 3 *C*, *broken line*) because the average distance between the linkers is increased due to fluctuations, which are especially pronounced in this case (Fig. 2 *C*). Overall, the FLD profile calculated for a mixture of T1 and T2 topoisomers (78) is in qualitative agreement with the experimental data (Fig. 3, *A*–*C*). Note similarity between the mutual positioning of gyres in Fig. 3, *E* and *F* and in Fig. 1, *E* and *F*. This implies that the results obtained for the fibers with NRL = 187 and 182 bp are also applicable to the other members of the T2 and T1 families.

In addition, Risca et al. (18) obtained genome-wide information on the intensities of the RICC-seq peaks, which reflect the spatial organization of chromatin fibers. This important information can be linked to the epigenetic maps of the active and repressed states of chromatin in different parts of the human genome. The active and the repressed chromatin regions are usually enriched with H3K27ac and H3K9me3 epigenetic marks, respectively (79). The relative FLD profiles calculated for these regions based on the results of Risca et al. (18) are presented in Fig. 3 *D*, along with the genome-wide FLD.

These curves are clearly different, with the H3K9me3-enriched profile having the highest amplitude at ∼370 nt and the H3K27ac profile having the lowest amplitude. Naturally, the genome-wide distribution has an intermediate profile. In a sense, the H3K9me3- and H3K27ac-enriched profiles are related in the same way as the theoretical MC profiles for the T2 and T1 topoisomers (Fig. 3, *B* and *C*). Indeed, in the T1 and H3K27ac-enriched profiles, the 370-nt peak is noticeably decreased compared to the T2 and H3K9me3 profiles.

These observations suggest that transcriptionally active and repressed genomic domains are characterized by different fractions of T1 and T2 topoisomers, the former clearly dominating in active domains enriched with the H3K27ac mark. This is consistent with our hypothesis that the novel T1 topoisomer (with L ≈ 10n+5) is associated with a high level of transcription (32,33).

In summary, the relative intensities of the FLD peaks observed by RICC-seq (18) provide important information on the spatial organization of chromatin fibers in various genomic regions; in particular, they can be used to distinguish between the topological states of chromatin.

## Conclusions

We have presented several lines of evidence for a topological polymorphism of chromatin fibers—in silico, in vitro, and in vivo. In addition to the well-known T2 topoisomer (46,47), we predicted (32,33) and later observed (59) a novel T1 family of forms. The two families differ by the level of DNA supercoiling (or linking number). The topological barrier makes interconversion between the T1 and T2 forms impossible without nicking-closing enzymes. Importantly, the T1 and T2 topoisomers are energetically favorable for different linker lengths (L ≈ 10n+5 and 10n, respectively). In other words, the nucleosome spacing defines the topological organization of chromatin (at least in vitro). Furthermore, the relative stability of these topoisomers is likely to depend on epigenetic histone modifications modulating the strength of internucleosome interactions (e.g., H4-K16-Ac). Potentially, observed correlations may reveal new mechanisms for encoding structural information in the form of alternative T1 and T2 topological states of nucleosome arrays.

The novel T1 topoisomer has a decreased level of DNA supercoiling (33) that is usually associated with active transcription (66). In addition, the T1 topoisomer is characterized by an increased plasticity, which makes chromatin more accessible to DNA binding factors and the RNA transcription machinery. Therefore, we suggested (33,34) that the {10n+5} DNA linkers produce transcriptionally competent chromatin structures, whereas the {10n} linkers may be important for the formation of stably folded chromatin fibers with high supercoiling typical of heterochromatin. This hypothesis is consistent with available data for yeast in which the nucleosome positioning shows regular {10n+5} and {10n} patterns downstream from the transcription start site (34) for the highly and lowly transcribed genes, respectively.

So far, the published topological studies of chromatin fibers were limited to the cases with regular nucleosome spacing (32, 33, 34, 35, 36). By contrast, the mesoscale modeling of chromatin (24,29) showed that the intrafiber NRL variations have a profound impact on chromatin structure, with a wide range of different architectures emerging, in agreement with high-resolution imaging data (9,13). We anticipate that by using our topological approach (33,34), it will be possible to evaluate changes in the DNA linking number, depending on the NRLs mixed.

Recent experiments have provided additional information on the polymorphic nature of chromatin fibers. First, it was found that the {10n+5} but not {10n} nucleosome arrays have a strong propensity for macroscopic self-association in vitro (19), which can be explained by more pronounced flexibility of the T1 topoisomer (Fig. 2).

Second, the genome-wide DNA cleavage induced by ionizing radiation, RICC-seq (18), was shown to reflect the spatial proximity of nucleosomes in chromatin fibers. The DNA cleavage pattern depends on the fiber configuration (i.e., T1 or T2); at the same time, it can be linked to the epigenetic maps of the active and repressed states of chromatin (Fig. 3).

Third, Micro-C (14, 15, 16) and Hi-CO (17) experiments revealed various structural motifs characterized by distinct nucleosome folding in vivo, from yeast to humans. Several distinct arrangements of nucleosomes were detected, two of which were denoted as IN-IN and OUT-OUT orientations (14), or “inward” and “outward” interactions of nucleosomes (17). These nucleosome folds occur in the T2 and T1 topoisomers, respectively (Fig. 1, *G* and *H*). Importantly, the “outward” interaction of nucleosomes (or the T1 fold in our classification) is prevalent in the transcribed regions of the yeast genome, according to Ohno et al. (see Fig. S3 H in (17)). A similar trend was observed for nucleosomes (i) and (i±2) in the OUT-OUT orientation, detected by Hsieh et al. (14) (D. Norouzi and V. B. Zhurkin, unpublished observation). These findings agree with the established correlation between formation of the T1 topoisomer and transcription in yeast (34).

Thus, we can say that despite the apparent discrepancy in notations used by different authors (14, 15, 16, 17), a new concept is emerging, establishing remarkable diversity of irregular nucleosome filaments having distinctive relative orientations of spatially close nucleosomes. Naturally, the structural and topological polymorphism of chromatin fibers is important from the point of view of genome regulation and maintenance.

We see the next goal in elucidating functional interrelations between the local folding motifs at the oligonucleosomal level and the global topological organization of chromatin, in terms of the linking number and superhelical density of DNA. It would be interesting to see whether the new findings reflect a more general tendency of chromosomal domains containing active or repressed genes (i.e., domains associated with different epigenetic marks) to retain topologically distinct higher-order structures.

## Author contributions

Both authors V.B.Z. and D.N. have conceived and written this review.
